# Extending VIVO Ontology to Represent Research and Educational Resources in an Academic Biomedical Informatics Department

**Published:** 2013

**Authors:** Drashko Nakikj, Chunhua Weng

**Affiliations:** Department of Biomedical Informatics, Columbia University, New York, NY, USA 10032

**Keywords:** information needs, ontology, semantic web, research networking system

## Abstract

The increasing need for interdisciplinary team sciences makes it vital for academic research departments to publicize their research and educational resources as part of “linked data” on the semantic web to facilitate research networking and recruitment. We extended an open-source ontology, VIVO, to represent the research and educational resources in an academic biomedical informatics department to enable ontology-based information storage and retrieval. Using participatory design methods, we surveyed representative types of visitors to the department web site to understand their information needs, and incorporated these needs into the ontology design. We added 114 classes and 186 properties to VIVO. Generalizability and scalability are the measures used in our theoretical evaluation.

## Introduction

Given the increasing need to facilitate interdisciplinary team science, many academic departments are motivated to share information about their research resources, including faculty expertise, students and alumni activities, edgy research areas, and training opportunities. Due to the vastness of the research field and its rapid changes, and the applied nature of biomedical informatics research topics, it is necessary but challenging for an academic department to attract right people from clinical environment, research settings, and industry to forge interdisciplinary collaborations and stay competitive. Taking Department of Biomedical Informatics (DBMI) at Columbia University as a case study example, information that is currently available on its web site is difficult for issuing semantic queries on, access it in an aggregated fashion and summarize content. It is also poorly reachable with high precision by external search engines. One potential solution to these problems is to develop an ontology to facilitate ontology-based information organization, retrieval, and sharing. A primary barrier is that there is a significant knowledge gap between existing research resource ontologies, represented by VIVO, and the rich content on our departmental web site. Therefore, the goal of this study is two fold: (1) to assess the information needs of visitors; and (2) to extend VIVO as needed to develop an ontology for annotating the research and educational resources of our departmental web site.

## Methods

Our design process closes the feedback loop between ontology users and designers and follows these three steps: (1) defining the scope of the resource ontology; (2) understanding the information needs of website visitors; and (3) assessing the information and knowledge gap using the current web site and VIVO ontology. We identified the typical website visitors and grouped them by roles, including scientists, students, organizations, e.g., companies, agencies, universities, and alumni. The same visitor can play multiple roles. To better determine the scope of the ontology and its purposes, we decided to survey two representative groups of visitors: students (role of current student and prospective student) and alumni (role of scientist and organization), both from our department, and understand their information needs. Students were asked to list up to 10 questions that they would like the website to provide information for. The survey for the alumni was omitted from this paper for the next development iteration. After we defined the scope of the ontology through analysis of the information needs, we used the existing DBMI website, interviewed the administration personnel, students, and faculty to get better and more detailed understanding of the domain and eliminate potential knowledge gaps.

## Results

We identified eight categories of information needs in various contexts: 1. information for people in the department (profiles, student advisor pairing) 2. general departmental overview (DBMI mission statement, overview and aims, difference from others) 3. information for prospective students (application, deadlines, contact person) 4. description of the educational programs (different study programs, trainee handbook, placement after graduation) 5. news and events (cutting edge research, instruction to set-up working environment, calendar with meetings and events) 6. department resources (departmental forms and deadlines, space and IT resources) 7. collaboration and activities (working on projects, job positions) 8. research areas and projects (seminal work, labs, research projects). We decided to use VIVO 1.4.1 as the basis for our ontology, because it already has some key classes important for the DBMI ontology: Person, Organization, InformationResource, Project, Event, Location, EducationalTraining, AdvisingRelationship. We added 114 classes and 186 properties to VIVO. The top-level classes we identified in the DBMI domain with respect to the scope are: DBMIPeople, DBMIEducation, DBMIResearchResource, DBMINews, DBMIEvent. Its reusability and generalizability is promising because it captures most of the common BMI department settngs and builds on VIVO. The scalability is yet to be evaluated in the next iteration of development (alumni survey), and granularity is very satsfying because it covers adequate details with depth.

## Conclusion

This paper represents the first step towards semantical modeling of the information content for CU DBMI web site and contributes empirical knowledge for designing an ontology for academic BMI department resources for the semantic web.

